# Methanol fixation and tagmentation of RNA/DNA hybrids directly enable single-cell transcriptome sequencing

**DOI:** 10.3389/fgene.2025.1629655

**Published:** 2025-07-30

**Authors:** Tao Xu, Yicong Xu, Ziyang An, Yiheng Li, Jiawen Yang, Weixing Zhang, Jin Xu

**Affiliations:** State Key Laboratory of Biocontrol, School of Life Sciences, Sun Yat-Sen University, Guangzhou, Guangdong, China

**Keywords:** methanol fixation, RNA/DNA hybrids, full-length transcriptome, scRNA-seq, RNA junctions

## Abstract

**Objective:**

Single-cell transcriptome sequencing is a powerful tool for investigating cellular diversity in normal development and disease. However, prevalent methods predominantly employ 3′-end sequencing of transcripts, limiting the analysis of alternative splicing and other post-transcriptional processes. While full-length single-cell transcriptome sequencing methods, such as Smart-seq, offer more comprehensive information, but are restricted by low-throughput. To overcome these limitations, we propose a strategy that combines *in situ* reverse transcription and transposition with a high-throughput micro-fluid platform to enable scalable full-length transcriptome profiling at single-cell resolution.

**Methods:**

In this study, we utilized methanol fixation on cultured cells to evaluate RNA integrity and cellular preservation post-fixation. *In situ* reverse transcription followed by RNA/DNA hybrids transposition was performed to test the efficiency of these reactions. The transposed fragments were sequenced and investigated to determine transcriptome capture efficiency. Finally, we combined *in situ* reactions with the 10X Genomics scATAC-seq platform to prepare a single-cell transcriptome library, aiming to assess the feasibility of full-length transcriptome sequencing at the single-cell level using this combined approach.

**Results:**

Methanol fixation enables preservation of RNA and facilitates *in situ* reverse transcription of full-length cDNA. Importantly, cells maintain their integrality after reverse transcription and transposition even under low concentration of methanol. Reducing the methanol concentration to 40% further enhances transcript capture efficiency. At the single-cell level, this strategy enables the capture of full-length transcriptomes, demonstrating a great potential for application in single-cell sequencing.

## 1 Introduction

The transcriptome serves as a fundamental molecular blueprint, defining cellular identity and reflecting the functional state of cells. Recent advances in single-cell RNA sequencing (scRNA-seq) have revolutionized transcriptomic analysis, enabling the dissection of gene expression heterogeneity across diverse cell types and tissues. These technologies provide unprecedented insights into cellular function, developmental trajectories, and disease pathogenesis (Klein et al., 2015; Macosko et al., 2015; Zheng et al., 2017).

However, constrained by the read-length limitations of second-generation sequencers, most current scRNA-seq methods, such as those employed by 10X Genomics platform, typically capture only the 3′- or 5′-end of each mRNA transcript following reverse transcription. While this approach enhances transcript stability and processing efficiency, it inherently limits the acquisition of full-length transcriptome information, thereby restricting analyses of isoform diversity, splicing events, and regulatory elements (Deng et al., 2014; Byrne et al., 2017).

To address this deficiency, plate-based methods such as Smart-seq have been developed and are now considered the “gold standard” for single-cell full-length transcript sequencing (Ramsköld et al., 2012; Picelli et al., 2014b; Hagemann-Jensen et al., 2020; Hagemann-Jensen et al., 2022). Despite their high sensitivity and accuracy, these methods necessitate cell sorting into 96- or 384-well plates, followed by separate processing of each well. This workflow is labor-intensive, cost-prohibitive, and therefore unsuitable for large-scale applications.

To overcome these limitations, we propose an alternative strategy involving *in situ* reverse transcription and transposition directly within intact cells, followed by single-cell separation and barcoding using micro-fluid devices. However, its feasibility remains uncertain, as the key steps—*in situ* reverse transcription and transposition—have not been systematically evaluated.

Tn5 transposase is widely employed for its ability to simultaneously fragment double-stranded DNA (dsDNA) and ligate sequencing adapters (Adey et al., 2010; Picelli et al., 2014a), making it a cornerstone tool in chromatin accessibility assays and dsDNA library preparation (Chen et al., 2016; Mumbach et al., 2016; Kaya-Okur et al., 2019). Recent studies have revealed that Tn5 transposase also exhibits activity on RNA/DNA hybrids, enabling direct tagmentation of RNA/DNA hybrids without the need for second-strand synthesis (Di et al., 2020; Lu et al., 2020; Xu et al., 2022; Li et al., 2023). This capability holds the potential to reduce biases introduced by incomplete reverse transcription and GC skew during amplification (Di et al., 2022). However, the activity of Tn5 transposase on RNA/DNA hybrids has not been assessed in the context of whole cells, where conditions are more complex than *in vitro*.

In this study, we employed methanol fixation for cultured cells and found that methanol-fixed cells not only protected RNA from degradation but maintained cellular structure, even under low-concentration conditions. We further evaluated *in situ* reverse transcription and transposition at both bulk and single-cell levels. Our results demonstrate that this strategy enables the capture of full-length transcripts and holds strong potential for high-throughput single-cell transcriptomic profiling.

## 2 Materials and methods

### 2.1 Cell culture

The GM12878 cells were cultured in RPMI 1640 medium (Gibco) supplemented with 10% fetal bovine serum (Corning) and 1% penicillin-streptomycin (Gibco). The HEK293T and NIH/3T3 cells were cultured in Dulbecco’s Modified Eagle Medium (DMEM) (Gibco) supplemented with 10% fetal bovine serum and 1% penicillin-streptomycin. Adherent cells were detached with 0.25% Trypsin-EDTA (Gibco) at 37°C for 3 min and quenched with growth medium. All cells were kept at 37°C with 5% CO_2_.

### 2.2 Cell fixation

For cell fixation steps, 1 million cultured cells were washed twice with 1 mL of ice-cold PBS and resuspended in 200 μL of ice-cold PBS. Subsequently, 800 μL of methanol (CST, pre-chilled to −20°C) was added dropwise and gently mixed. Methanol fixation was performed at −20°C for 30 min. Fixed-cells were pelleted at 500 × g for 5 min at 4°C. The cell pellet was washed twice with 1 mL of ice-cold Wash-resuspension buffer I (0.04% (w/v) BSA (Miltenyi Biotec), 1 mM DTT (Thermo Fisher) and 2 U/μL RiboLock RNase Inhibitor (Thermo Fisher)). Finally, the permeabilized cells were resuspended in 20 μL–50 μL of ice-cold Wash-resuspension buffer II (1% (w/v) BSA, 5 mM DTT, 2 U/μL RiboLock RNase Inhibitor) for downstream processing.

### 2.3 *In situ* reverse transcription

For the annealing and reverse transcription steps, cells were stained with trypan blue and counted for concentration. 10,000 methanol-fixed cells (about 1 μL) were mixed with 1 μL of 10 mM dNTP (Thermo Fisher) and 1 μL of 25 μM oligo-dT_30_VN and incubated at 55°C for 5 min, then immediately placed on ice for 2 min to facilitate RNA denaturation. Next, reverse transcription was performed by adding 7 μL RT mix (100 U maxima H minus reverse transcriptase (Thermo Fisher), 1.43 × reaction buffer, 7.14 mM DTT, 0.83 μM TSO, 20 U RiboLock RNase Inhibitor) and incubating for 10 min at 50°C, following by 3 cycles of (12 s at 8°C, 45 s at 15°C, 45 s at 20°C, 30 s at 30°C, 2 min at 42°C, and 3 min at 50°C), and concluding with 5 min at 50°C.

### 2.4 Full-length cDNA amplification and quality control

After reverse transcription, cells were further lysed by adding 5 μL Proteinase K (Sangon Biotech) and incubating at 56°C for 30 min and 95°C for 10 min. Subsequently, 45 μL PCR buffer, containing 2.5 μL 10 μM IS-PCR primer, 30 μL KAPA HiFi HotStart Ready Mix (KAPA) and 12.5 μL Nuclease-free water (NEB) were added. The PCR reaction was performed for 3 min at 98°C, following by 18 cycles of (20 s at 98°C, 15 s at 67°C, 6 min at 72°C), and concluding with 5 min at 72°C. The PCR product was purified by DNA Clean and Concentrator™-5 kit (Zymo Research) and the size distribution was tested by Standard Cartridge Quantitative Kit (Bioptic). All the primers in this section were adapted from Picelli et al. (2014b).

### 2.5 *In situ* full-length transcriptome library preparation

One million cultured cells (GM12878, HEK293T or NIH/3T3) were washed twice with 1 mL of ice-cold PBS and fixed in 1 mL ice-cold diluted methanol (methanol in PBS, methanol with different concentrations) at −20°C for 30 min. After two additional washes with 1 mL of ice-cold Wash-resuspension buffer I, permeabilized cells were resuspended in 20 μL–50 μL of ice-cold Wash-resuspension buffer II. Then, *in situ* reverse transcription was carried out.

After reverse transcription, 40 μL of Tagmentation buffer (12.5 mM Tris-HCl (pH 7.4) (Sigma), 6.25 mM MgCl_2_ (Sigma), 12.5% N, N-Dimethylformamide (Sigma), 11.25% PEG8000 (Beyotime), 1.06 mM ATP (NEB), 1 μL TTE Mix V50 (Vazyme)) was mixed with the RT product and the tagmentation reaction was performed at 37°C for 30 min.

Subsequently, cells were lysed by adding 5 μL of Proteinase K at 56°C for 30 min. The DNA Clean and Concentrator™-5 kit was used to purify the tagmented fragments and index PCR was carried out by adding index primers and Next High-Fidelity 2X PCR Master Mix (NEB). PCR was cycled as follows: 5 min at 72°C for gap filling, 1 min at 98°C for initial denaturation, following by 23 cycles of (15 s at 98°C, 30 s at 63°C, 1 min at 72°C), and 5 min at 72°C for final extension. The index primers used in this study were provided in Supplementary Table S1.

Following PCR, the indexed products were placed on a magnet and eluted into a new tube. Then, 0.6 volumes (30 μL) of VAHTS DNA Clean Beads (Vazyme) were mixed and incubated for 5 min at room temperature. The samples were placed on a magnet for 1 min, and the supernatant was transferred to a new tube. 0.2 volumes (10 μL) of VAHTS DNA Clean Beads were mixed and incubated for 5 min at room temperature. The samples were then placed on a magnet for 1 min, and the supernatant was discarded. After two additional washes with 200 μL 80% ethanol, samples were eluted with 20 μL Nuclease-free water. Subsequently, libraries were quantified by Qubit according to the manufacturer’s instruction. The libraries were sequenced with Illumina NovaSeq 6,000 by PE150 model.

### 2.6 Single-cell full-length transcriptome library preparation

For species-mixing experiment of single-cell full-length transcriptome library (scFL), 0.5 million human HEK293T cells and 0.5 million murine NIH/3T3 cells were mixed and pelleted. Cells were washed twice with 1 mL of ice-cold PBS and resuspended in 600 μL of ice-cold PBS. 400 μL of methanol (pre-chilled to −20°C) was added dropwise and gently mixed. Methanol fixation was performed at −20°C for 30 min. After two additional washes with 1 mL of ice-cold Wash-resuspension buffer I, permeabilized cells were resuspended in 20 μL–50 μL of ice-cold Wash-resuspension buffer II. Counting the cell concentration and diluting to 10,000 cells/μL with ice-cold Wash-resuspension buffer II.

32 PCR tubes were prepared and 1 μL cell suspension, 1 μL 10 mM dNTP and 1 μL 25 μM oligo-dT_30_VN were mixed in each tube. Incubated for 5 min at 55°C, and then placed on ice immediately for 2 min. 7 μL RT mix was added in each tube for *in situ* reverse transcription.

Then, adding 40 μL of PBS-1% BSA per tube, mixing gently, and pooling all the samples into a 15 mL tube (pre-blocked overnight with 1% BSA). The samples were pelleted at 500 × g for 5 min at 4°C. The PCR tubes were washed with additional 50 μL of PBS-1% BSA for maximum recovery. The supernatant was carefully removed, and 1 mL of 0.1% formaldehyde (Thermo Fisher) was used to fix the cells, preventing cell rupture and reducing RNA contamination during tagmentation and droplet encapsulation.

Cell pellets were resuspended in 10 μL–50 μL Diluted Nuclei Buffer (10X genomics). Staining cells with trypan blue, assessing cell morphology, and counting cell concentration. 15,000 cells were used for transposition. The procedure of transposition followed the manufacturer recommended protocol.

The procedures of GEM formation and barcoding, cleanup, index PCR and size selection were adapted from 10X genomics Chromium Next GEM Single Cell ATAC Reagent Kits v2 following manufacturer recommended protocols. After size selection, the library was quantified by Qubit according to the manufacturer’s instruction. The library was sequenced with Illumina NovaSeq 6,000 by PE150 model.

### 2.7 Preprocessing, alignment, and quantification of the bulk libraries and bulk RNA-seq libraries

For bulk full-length transcriptome libraries generated under different concentrations of methanol fixation, as well as for the corresponding bulk RNA-seq datasets (GM12878, HEK293T and NIH/3T3), raw data of the bulk RNA-seq libraries were obtained from the GEO repository (GSE30400, GSE205869 and GSE196318). The raw data of the GM12878 single-cell 3′-end RNA-seq library was obtained from the GEO repository (GSE126321) and the raw data of the HEK293T and NIH/3T3 single-cell 3′-end RNA-seq libraries were obtained from 10X official datasets as the pseudo-bulk dataset, respectively. Adapter sequences and low-quality reads (average sequencing quality less than Q15 or effective length less than 15 bp) were removed using fastp (Chen S. et al., 2018). Processed reads of GM12878 and HEK293T cells were aligned to the human reference genome (GRCh38.p13) using STAR (Dobin et al., 2013), while the reads of NIH/3T3 cells were aligned to the mouse reference genome (GRCm39). Duplicate reads with identical sequences were removed using Picard. Gene annotation was performed using the GENCODE v40 annotation file for human cell lines and GENCODE vM29 for mouse. Transcripts assembly and quantification were conducted using HTSeq (Putri et al., 2022) to calculate TPM values for each gene.

### 2.8 Preprocessing, alignment and quantification of the single-cell full-length transcriptome library

For the scFL dataset containing a mixture of HEK293T and NIH/3T3 cells, as well as for other mixture libraries used for comparison (GSE108097 and 10X official dataset), raw reads were pre-processed to removal adapter sequences and low-quality reads, following the same procedure as applied to the bulk RNA-seq data. Processed reads were aligned to both the human GRCh38.p13 and mouse GRCm39 reference genomes using STAR and reads mapped to both genomes were removed. Duplicate reads sharing identical barcodes and sequences were removed using Picard. Gene annotation was performed using GENCODE v40 for human and GENCODE vM29 for mouse. The number of reads mapped to exonic regions were calculated for each gene to generate the gene expression matrix.

### 2.9 Cell calling of the single-cell full-length transcriptome library

Utilizing the gene expression matrix, the total counts of each droplet were sorted, and one-tenth of the counts at the 100th rank were chosen as the threshold for removing empty droplets. Droplets with total counts of HEK293T genes exceeding 1,000 and a percentage of NIH/3T3 counts less than 0.25 were classified as HEK293T cells. Similarly, droplets with total counts of NIH/3T3 genes exceeding 1,000 and a percentage of HEK293T counts less than 0.25 were identified as NIH/3T3 cells. Droplets with a count percentage greater than 0.25 but a total count less than 30,000 were classified as contaminated cells. The remaining cells were identified as filtered cells, including low-quality cells and doublets. The selected cells were then subjected to principal component analysis and UMAP to extract expression features, which were compared with the cell classification results.

### 2.10 Identification of junctions and quantification of transcripts from the single-cell full-length transcriptome library

Reads corresponding to HEK293T and NIH/3T3 cells were independently extracted from the scFL dataset. Splice junctions within each gene were classified and quantified based on the previously mentioned annotation. These extracted reads were subsequently subjected to transcript assembly and quantification using HTSeq. For alternative splicing analysis and single-cell transcript quantification, genes were selected based on the following criteria: (1) presence of skipped exons (SE) events, (2) a proportion of the same transcript type exceeding 50% as compared to the bulk RNA-seq library, (3) fewer than 5 reads supporting the same transcript type, and (4) expression levels (TPM) exceeding 16.

### 2.11 Comparison of gene and transcript expression correlations between datasets

Genes and transcripts expression levels were quantified using the number of transcripts per million (TPM). Pearson correlation coefficient and statistical significance were calculated using R.

## 3 Results

### 3.1 Enabling *in situ* reverse transcription through methanol-based cell fixation

**FIGURE 1:**
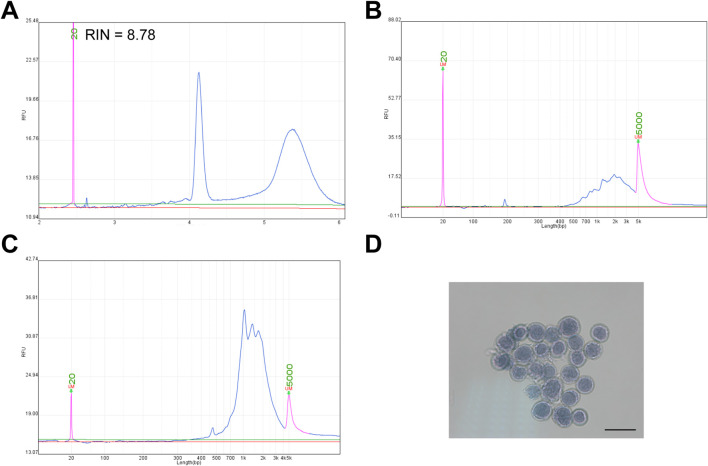
Methanol fixation does not impair *in situ* reverse transcription in GM12878 cells. **(A)** RNA quality of GM12878 cells following methanol fixation (RIN, RNA integrity number). **(B)** Size distribution of cDNA generated by *in vitro* reverse transcription of RNA from methanol-fixed GM12878 cells. **(C)** Size distribution of cDNA generated by *in situ* reverse transcription of methanol-fixed GM12878 cells. **(D)** Microscopic image of GM12878 cells stained with trypan blue following *in situ* reverse transcription, scale bar: 20 μm.

Methanol is widely used for cell fixation and permeabilization due to its dehydrating properties and lipid solubility. In this study, we fixed cultured GM12878 cells with 80% methanol, and subsequently extracted total RNA. RNA quality assessment indicated that the RNA was well preserved, with no significant degradation, consistent with previous studies (Figure 1A) (Chen J. et al., 2018; Wang et al., 2021). Since methanol disrupts the cell membrane, it facilitates the entry of macromolecules, such as antibodies, into the cells. Additionally, methanol-induced protein denaturation and precipitation may enhance mRNA accessibility by reducing physical barriers to *in situ* reverse transcription. Based on this hypothesis, we performed reverse transcription directly in 80% methanol-fixed cells. As a control, total RNA extracted from cells fixed with 80% methanol was subjected to conventional *in vitro* reverse transcription. The cDNA size distribution was comparable between the *in vitro* and *in situ* approaches (Figures 1B,C). Importantly, cell morphology remained intact following *in situ* reverse transcription (Figure 1D).

To evaluate the broader applicability of this assay, we extended the strategy to HEK293T and NIH/3T3 cell lines. Consistent with observation in GM12878 cells, methanol fixation did not compromise RNA quality in either HEK293T or NIH/3T3 cells (Supplementary Figures 1A,D), and full-length transcripts were successfully captured after *in situ* reverse transcription (Supplementary Figures 1B,E). Additionally, the cellular morphology was preserved throughout the process, as observed under the microscope (Supplementary Figures 1C,F).

Collectively, these results demonstrate that methanol-fixed cells are compatible with *in situ* reverse transcription of full-length cDNA, while maintaining cellular morphological.

### 3.2 Integration of *in situ* reverse transcription and hybrid transposition enables full-length transcriptome profiling

**FIGURE 2:**
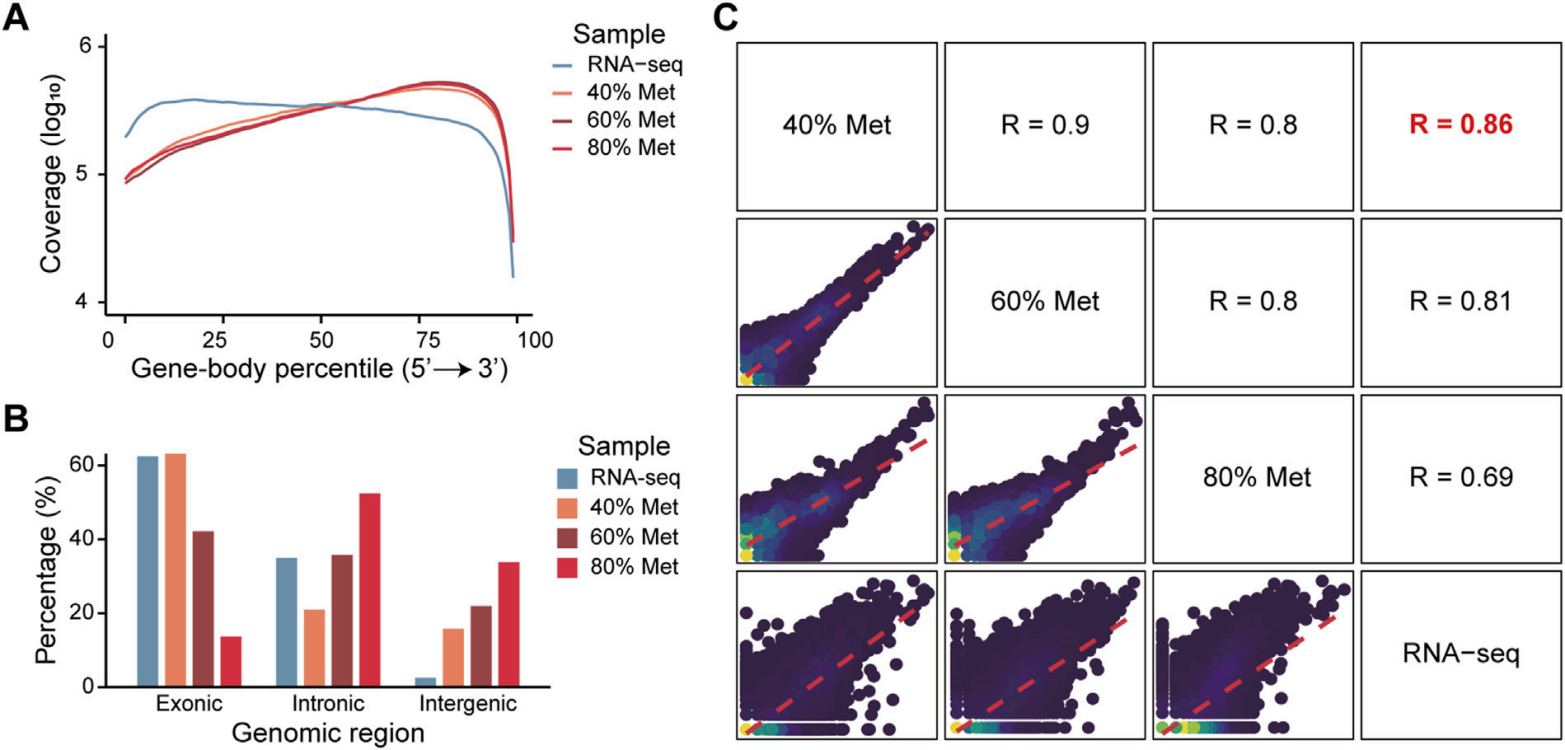
Quality assessment and effects of methanol concentrations on transcriptome capture efficiency. **(A)** Gene-body coverage profiles of libraries prepared with various methanol concentrations. Mean coverage at each gene body position was normalized by the total sequencing depth of each sample using log_10_ transformation. Colors indicate different methanol concentrations. **(B)** The proportion of reads mapped to different genomic regions across samples fixed with various methanol concentrations. Colors indicate the different methanol concentrations for each sample. **(C)** Pairwise comparison of gene expression profiles between samples fixed with different methanol concentrations and corresponding bulk RNA-seq data. Scatter plots in the lower left panels display the consistency of log_10_ transformed gene expression levels across conditions.

To further evaluate the feasibility of *in situ* transcriptome profiling, we employed Tn5 transposase to tagment RNA/DNA hybrids generated by reverse transcription in methanol-fixed cells. The Tn5 tagmentation was performed *in situ,* followed by PCR amplification based on a protocol adapted from ATAC-seq (Buenrostro et al., 2013). To validate the success of *in situ* tagmentation of full-length cDNA, we sequenced the PCR products and compared the resulting transcriptome profiles with those obtained from conventional bulk RNA-seq. As expected, the sequence data demonstrated typical full-length gene body coverage (Figure 2A), with a slight 3′ bias. These findings confirmed that full-length reverse transcription and subsequent transposition of RNA/DNA hybrids can be effectively achieved *in situ* in methanol-fixed cells.

Given that Tn5 transposase exhibits higher activity toward dsDNA than RNA/DNA hybrids, genomic DNA contamination may interfere with accurate transcriptome profiling. Based on the differing phospholipid compositions of the cytomembranes and nuclear membranes, we hypothesized that lower methanol concentrations might selectively permeabilize the cytomembranes without disrupting the nuclear membranes, thereby minimizing the Tn5 transposase access to nucleus. To test this hypothesis, we fixed cells with varying concentrations of methanol and evaluated their effects on transcriptome profiling. As expected, lower methanol concentrations did not compromise RNA quality (Supplementary Figures 2A,D) and *in situ* reverse transcription efficiency (Supplementary Figures 2B,E), and preserved overall cell structure (Supplementary Figures 2C,F). Intriguingly, a gradual increase in the fraction of reads mapped to exonic regions was detected, while full-length transcript coverage was maintained (Figures 2A,B). At 40% methanol concentration, the reads distribution on the genome closely resembled that of the bulk RNA-seq dataset (Figure 2B), and the transcriptome data showed the highest correlation with the bulk RNA-seq dataset (Figure 2C). Overall, our full-length transcriptome sequencing approach (FL) demonstrated a high degree of consistency when compared with 3′-end RNA-seq and standard RNA-seq, indicating that our method yields robust and reliable expression quantification comparable to established RNA-seq platforms (Supplementary Figure 3).

These findings suggest that combining methanol fixation, *in situ* reverse transcription, and Tn5 transposase mediated RNA/DNA hybrids transposition enables efficient capture of full-length transcriptome. Moreover, optimizing methanol concentration, particularly using lower concentrations, such as 40%, can improve transcript capture without compromising coverage or cellular integrity.

### 3.3 Leveraging *in situ* reverse transcription and hybrid transposition for droplet-based single-cell full-length transcriptomics

**FIGURE 3:**
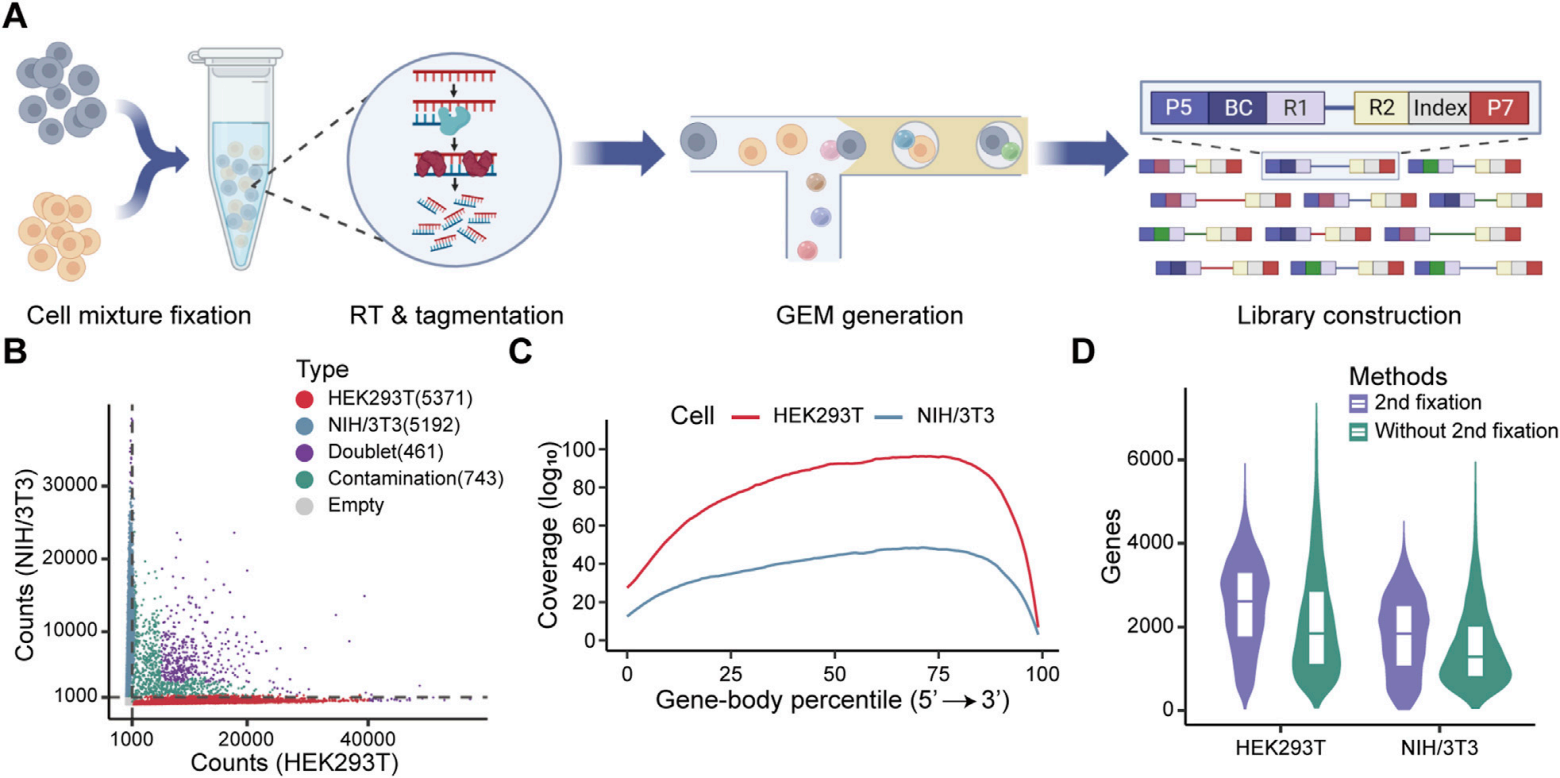
Evaluation of full-length transcriptome profiling at the single-cell level using formaldehyde for secondary fixation. **(A)** Schematic diagram of the single-cell library construction workflow. **(B)** Classification of droplets containing HEK293T and NIH/3T3 cells based on transcript counts. Droplets were classified into five categories (HEK293T cells, NIH/3T3 cells, doublet cells, contaminated cells, and empty) based on the threshold of 1,000 counts (gray dashed line) and the percentage of counts, with each category represented by a distinct color. The data following each category indicates the number of cells. **(C)** Gene body coverage for each cell type following secondary fixation with formaldehyde. The log_10_ transformed mean coverage at each gene body site was normalized by the number of cells. Colors represent different cell types. **(D)** Comparation of the number of detected genes in HEK293T and NIH/3T3 cells with or without formaldehyde secondary fixation.

Following reverse transcription, the cells maintained their structure integrity, and subsequent morphological assessment after tagmentation showed no apparent damage (Supplementary Figure 4A). Notably, the ability of RNA/DNA hybrids tagmentation to directly profile full-length transcripts highlights the potential for droplet-based single-cell full-length transcriptome profiling. This can be achieved by integrating *in situ* reverse transcription and tagmentation steps with the 10X Genomics platform, specifically utilizing the single-cell ATAC-seq kit. The workflow consists of four key steps: (1) fixing cells with a low concentration of methanol to permeabilize the cytomembrane while preserving cellular structure for *in situ* reverse transcription; (2) performing *in situ* tagmentation of RNA/DNA hybrids using Tn5 transposase; (3) separating and indexing single cells using the Chromium Next GEM Single Cell ATAC-seq Kit, and (4) sequencing and analyzing the full-length transcriptome (Figure 3A).

We applied this protocol to a mixture of human HEK293T cells and mouse NIH/3T3 cells. The cell mixture was fixed and permeabilized with methanol, followed by *in situ* reverse transcription, Tn5 tagmentation, and single-cell library preparation. Methanol fixation and *in situ* reactions did not affect droplet formation (Supplementary Figure 4B). However, after filtering out low-quality cells, only 5,330 high-quality cells were recovered in a single run (Supplementary Figure 4C), which is far below than the expected number of captured cells (∼10,000 cells). We speculated that the cells may be ruptured during GEM formation, as indicated by a high rate of cross-species contaminations (Supplementary Figure 4C).

To address this issue, we introduced a secondary fixation step using formaldehyde prior to transposition. This modification significantly improved performance. We recovered 10,563 high-quality cells and observed a marked reduction in cross-species contamination (Figure 3B). Furthermore, this optimized protocol not only enabled the robust capture of full-length transcripts, but also substantially improved gene-body coverage (Supplementary Figures 3C,4D). Formaldehyde fixation also enhanced the number of detected genes per cell in both HEK293T and NIH/3T3 cells (Figure 3D). These results demonstrate that formaldehyde-based secondary fixation can effectively improve single-cell recovery and gene detection efficiency.

We further evaluated the performance of this single-cell full-length transcriptome assay (scFL) with secondary fixation. Cells were accurately assigned to their respective species based on their transcriptome characteristic and genome mappability (Supplementary Figures 4E,F). We then benchmarked this data against Microwell-seq2, a recently developed cost-effective and high-throughput single-cell sequence protocol (Chen et al., 2021), as well as against standard 10X Genomics scRNA-seq datasets generated from HEK293T and NIH/3T3 cells. Our method detected a comparable number of genes per cell across both cell types (Supplementary Figures 4G,H). Moreover, technical replicates exhibited high consistency, demonstrating the reproducibility of the method (Supplementary Figure 4I). In addition, secondary fixation improved the correction of gene expression quantification with both standard 10X Genomics scRNA-seq and bulk RNA-seq across both cell types, supporting the enhanced accuracy and reliability of transcript quantification achieved by our approach (Supplementary Figure 4J).

### 3.4 Evaluation of junctions in droplet-based single-cell full-length transcriptomics

**FIGURE 4:**
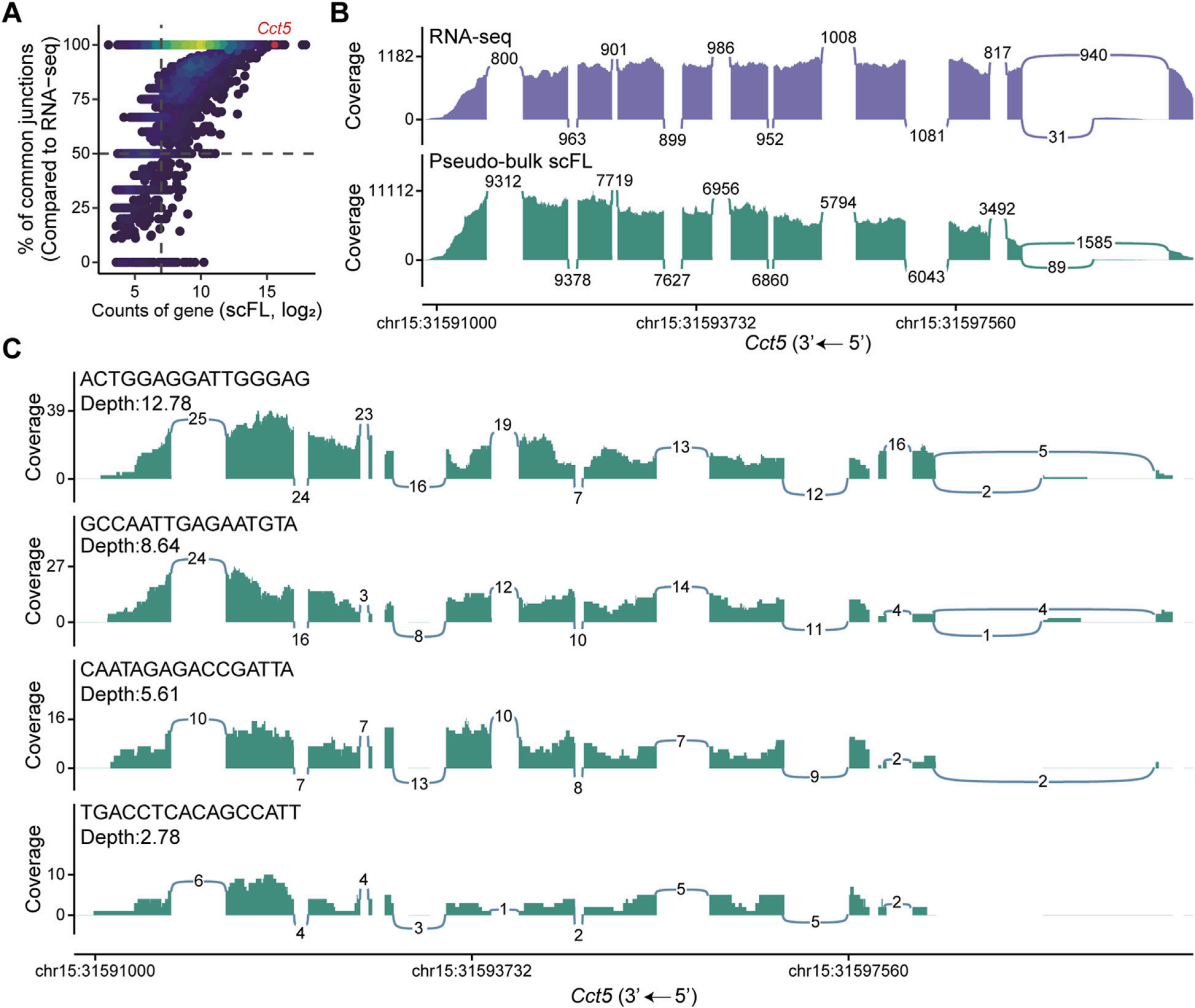
Quantification of splicing junctions in NIH/3T3 cells. **(A)** Correlation between gene expression levels and the proportion of isoforms with identical splice junctions shared between the pseudo-bulk scFL library and corresponding bulk RNA-seq dataset from NIH/3T3 cells. **(B)** Visualization of alternative splicing in the *Cct5* gene based on pseudo-bulk scFL and bulk RNA-seq dataset from NIH/3T3 cells. The height of the graphs indicates relative coverage at each genomic position, and the numbers in the plot indicate the count of reads supporting the splice junctions. **(C)** Alternative splicing of the *Cct5* gene at the single-cell level across different sequencing depths. Cell barcodes are shown in the top left of each panel.

To evaluate the performance of our single-cell full-length transcriptome dataset beyond gene expression, we further examined its ability to detect exon-junctions, a critical criterion for evaluating isoform divergence using short-reads sequencing strategies. We first aggregated the single-cell data into a pseudo-bulk transcriptome and compared the splicing junction profiles with those derived from standard bulk RNA-seq data. Isoform counts for each gene were subsequently estimated based on junction usage. This analysis revealed high concordance between the pseudo-bulk and bulk datasets (Supplementary Figure 5A). At a global level, the junction detection rate increased with both gene expression levels and total sequencing depth, suggesting transcript abundance and sequencing coverage jointly influence the sensitivity of splicing event detection (Figure 4A; Supplementary Figure 5B). We then focused on genes with multiple isoforms and quantified their splicing events, benchmarking our results against those from conventional bulk RNA-seq. The sashimi plot demonstrated that our method not only successfully detected all expected junction types, but also reproduced the proportional representation of these junctions, enabling reliable identification of alternative splicing events (Figure 4B; Supplementary Figure 5C). To further investigate splicing detection at the single-cell level, we selected individual cell with varying sequencing depths and visualized their coverage of splicing events. As expected, cells with enough sequencing depth captured all major splicing events, confirming the method’s capacity to resolve isoform diversity at single-cell resolution (Figure 4C; Supplementary Figure 5D).

In summary, our approach employs methanol fixation for effective single-cell library preparation, preserves both cellular morphology and transcriptomic integrity. The combination of *in situ* reverse transcription with RNA/DNA hybrid tagmentation enables comprehensive full-length transcriptome profiling at single-cell resolution. This method provides a scalable and accessible solution for high-throughput single-cell full-length transcriptomic analysis, providing valuable insights into gene expression and alternative splicing in a more accessible and scalable manner. Furthermore, our systematical evaluation and optimization of each process step, including the use of low-concentration methanol to improve transcriptome capture efficiency and formaldehyde fixation prior to transposition to enhance cell recovery and gene detection efficiency, paves the road for future research.

## 4 Discussion

In this study, we demonstrated that methanol-fixed cells enable *in situ* full-length reverse transcription without compromising cellular structure. Building on this finding, we combined *in situ* full-length reverse transcription with RNA/DNA hybrids transposition to evaluate the feasibility of full-length transcriptome profiling at both bulk and single-cell levels. Our results indicate that using a lower concentration of methanol contributes to improving transcription capture efficiency, while a secondary fixation step with formaldehyde significantly increases cell recovery. Together, these reagent combinations present a promising strategy for single-cell full-length transcriptome mapping.

Despite the promise of this strategy, several challenges remain in establishing a robust method for single-cell full-length transcript sequencing. The most critical aspect of transcriptome sequencing is maintaining RNA integrity. Although we have successfully achieved full-length cDNA distribution following *in situ* reverse transcription in certain cultured cells, it remains difficult to preserve intact RNA in some primary cells, which often contain higher level of proteases and RNases (Chen J. et al., 2018). RNA degradation occurs rapidly upon rehydration, suggesting that further optimization will be necessary to protect RNA during early processing steps. Additionally, to maintain cellular morphology, conventional PCR-based cDNA amplification is not suitable, as the high thermal cycling conditions can damage cell structure. Although we employed Tn5 transposase to directly transpose RNA/DNA hybrids, circumventing the need for second-strand synthesis, this approach exhibited reduced sensitivity for detecting low-expressed genes, particularly at limited sequencing depths (Figure 4A; Supplementary Figure 5B). To address this limitation, incorporating isothermal amplification techniques, such as rolling circle amplification (RCA) or *in vitro* transcription (IVT), may offer effective alternatives for enhancing sensitivity without compromising cell integrity (Ke et al., 2013; Fandrey et al., 2024).

## Data Availability

The raw data and annotated count matrices for both bulk and single-cell full-length transcriptome are publicly accessible in the National Center for Biotechnology Information (NCBI) Gene Expression Omnibus (GEO) under the accession number GSE233170. Code used for the analysis of bulk and single-cell data, as well as for the generation of all figures, can be accessed at https://github.com/0CBH0/FLITseq_pipeline.
